# Caucasian Blueberry: Comparative Study of Phenolic Compounds and Neuroprotective and Antioxidant Potential of *Vaccinium myrtillus* and *Vaccinium arctostaphylos* Leaves

**DOI:** 10.3390/life12122079

**Published:** 2022-12-11

**Authors:** Arnold A. Shamilov, Daniil N. Olennikov, Dmitryi I. Pozdnyakov, Valentina N. Bubenchikova, Ekaterina R. Garsiya, Mikhail V. Larskii

**Affiliations:** 1 Department of Pharmacognosy, Botany and Technology of Phytopreparations, Pyatigorsk Medical-Pharmaceutical Institute, Branch of Volgograd State Medical University, Ministry of Health of Russian Federation, 11 Kalinina Avenue, 357500 Pyatigorsk, Russia; 2 Laboratory of Biomedical Research, Institute of General and Experimental Biology, Siberian Division, Russian Academy of Science, 6 Sakhyanovoy Street, 670047 Ulan-Ude, Russia; 3 Department of Pharmacology and Clinical Pharmacology, Pyatigorsk Medical-Pharmaceutical Institute, Branch of Volgograd State Medical University, Ministry of Health of Russian Federation, 11 Kalinina Avenue, 357500 Pyatigorsk, Russia; 4 Department of Pharmacognosy and Botany, Kursk State Medical University, Ministry of Health of Russian Federation, 3 Karl Marks Street, 305000 Kursk, Russia; 5 Department of Pharmaceutical Chemistry, Pyatigorsk Medical-Pharmaceutical Institute, Branch of Volgograd State Medical University, Ministry of Health of Russian Federation, 11 Kalinina Avenue, 357500 Pyatigorsk, Russia

**Keywords:** blueberry, *Vaccinium myrtillus*, *Vaccinium arctostaphylos*, phenolic compounds, antioxidant activity, neuroprotective activity

## Abstract

(1) Background: Two Caucasian blueberries *Vaccinium myrtillus* L. and *Vaccinium arctostaphylos* L. are famous berry bushes growing in the Caucasus region and used to treat neurological diseases, but the chemistry and bioactivity of leaf extracts are still poorly studied. (2) Methods: Phenolic compounds of *V. myrtillus* and *V. arctostaphylos* leaf extracts were profiled and quantified by HPLC–PDA–ESI–tQ–MS. The neurotropic potential of *Vaccinium* extracts was studied using the model of middle cerebral artery permanent occlusion to determine cerebral blood flow, the area of the brain tissue necrosis, and antioxidant enzyme activity (including superoxide dismutase, succinate dehydrogenase, and cytochrome C oxidase), as well as the concentration of TBARS. (3) Results: Hydroxycinnamates and flavonoids were identified in the leaves of *V. myrtillus* and *V. arctostaphylos*, and the dominant metabolite of both extracts was 5-*O*-caffeoylquinic acid in the amount of 105–226 mg/g. The studied extracts enhanced the cerebral hemodynamics and decreased the frequency of necrotic and lipooxidative processes in the brain tissue, accompanied by an increase in the activity of antioxidant enzymes. The positive effect of *V. arctostaphylos* was stronger and exceeded the effectiveness of *Ginkgo biloba* standardized extract. (4) Conclusion: The leaf extracts of Caucasian blueberries *V. myrtillus* and *V. arctostaphylos* as a new source of hydroxycinnamates demonstrated a protective effect of the brain ischemia pathology and can be used as therapeutic agents to treat neurological diseases.

## 1. Introduction

Currently, the *Vaccinium* L. genus includes over 170 species [1], and there are 14 native species in the Russian Federation, including European blueberry (*V. myrtillus* L.), which grows throughout Europe outside of Central Asia, and Caucasian blueberry (*V. arctostaphylos* L.), which is an endemic plant of Transcaucasia and North Caucasus [2,3]. The fruits of *V. myrtillus* are included in pharmacopoeias of different countries as an astringent, hypolipidemic, and vision-improving remedy [4]. It is also known that an aqueous decoction of the fruit has antiprotozoal, antitumor, and cytotoxic activity [5,6,7]. Literature data indicate that *V. myrtillus* is a rich source of phenolic acids [8,9], flavonoids, and phenolic glycosides, as well as triterpenoids, carotenoids, organic acids, carbohydrates, and higher fatty acids [10,11].

**Figure 1:**
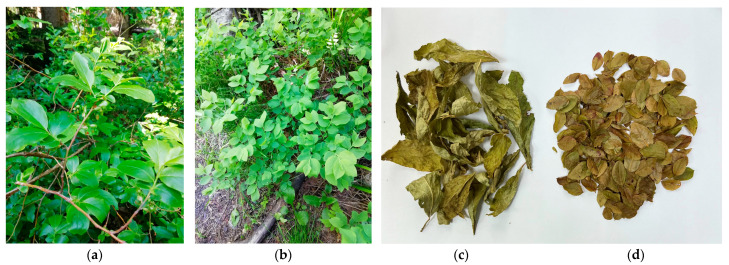
*Vaccinium* spp. in their natural habitat: *Vaccinium arctostaphylos* L. (Caucasian blueberry, bank of Belaya river, Khamyshki village, Maykop District, Republic of Adygea) (**a**); *Vaccinium myrtillus* L. (bank of the Cherek River, Kabardino–Balkarian Republic) (**b**). Dried leaves of *V. arctostaphylos* (**c**) and *V. myrtillus* (**d**).

In contrast to the European blueberry, *V. arctostaphylos* is not a pharmacopoeial plant due to insufficient knowledge of its chemical composition and pharmacological activity. Botanically, the Caucasian blueberry is a shrub or small tree up to 2–3 m high with rounded branches and large oblong leaves. Flowers in the raceme and the corolla are large whitish red. Fruits are large, globular, and black with a smooth surface. The leaves of *V. arctostaphylos* are up to 10 cm length compared to the leaves of *V. myrtillus*, which are up to 3 cm (Figure 1). To date, the chemical composition of fruits was only slightly studied [12,13], and there is a need for new studies on this promising source of bioactive metabolites. Additionally, some phenolics have been identified in the leaves [14], and hypoglycemic and hypotensive activities have been demonstrated for the leaf extracts [15,16].

Phenolics have a significant effect on the human organism and are primarily used as effective remedies for preventing a wide range of diseases from atherosclerosis to ischemic stroke. Phenolic compounds and total phenolics-based phytocompositions are increasingly used in modern neurology practice, for example, for the treatment and prevention of cerebral ischemia and neurodegenerative diseases [17,18]. Previously, it has been shown that the addition of polyphenols to the diet contributes to the development of a number of therapeutic benefits in patients with Parkinson’s disease, apparently by reducing oxidative damage of the *substantia nigra* neurons [19]. In addition, a number of studies show that the administration of polyphenolic substances significantly reduces damage to neurons during ischemia [20]. Moreover, polyphenolic substances are often considered as compounds that eliminate the hyperproduction of ROS and oxidative stress [21,22], and their neuroprotective effects have been clinically confirmed [21]. The results of a prospective cohort study by Gao et al. demonstrated a significant reduction in the risk of stroke when large amounts of dietary polyphenols are consumed, particularly citrus fruits and grapes [23]. Of note, some gender differences were identified in this work: the pharmacological efficacy of polyphenols was higher in women than in men [23].

Disorders of cerebral circulation represent a considerable medical and social problem. According to the World Health Organization, stroke ranks second among the causes of mortality and first among the causes of primary disability [24]. At the same time, approximately 80% of all stroke cases are due to ischemic brain damage [25]. One of the therapeutic options for the adjuvant treatment of cerebral ischemia is neuroprotection, in which a certain share is provided by herbal medicines. For example, some traditional Chinese medicine products have a pronounced neuroprotective effect, and the polyvalence of their action is important, affecting many pathogenetic mechanisms of cellular damage in ischemia: from oxidative stress to metabolic homeostasis and excitotoxicity [26]. 

The genus *Vaccinium* L., along with classical plant sources of antioxidants, can serve as a basis for the search for new biologically active compounds with neuroprotective activity; for example, the neuroprotective properties of anthocyanins of blueberries have been established [27]. In leaves, the main phenolic groups are proantocyanidines, flavonols, and hydroxycinnamic acids with potential activity in therapy of Alzheimer’s and Parkinson’s disease [28].

As part of the ongoing study of *Vaccinium* species [29,30], in this study, the phenolic compounds from *V. myrtillus* and *V. arctostaphylos* leaves were profiled and quantified by HPLC–PDA–ESI–tQ–MS assay, and the antioxidant and neuroprotection potential of total blueberry leave extracts was estimated in brain ischemia in vivo experiments.

## 2. Materials and Methods

### 2.1. Plant Material and Chemicals

Samples of *Vaccinium myrtillus* L. leaves were collected on the bank of the Cherek River, Kabardino–Balkarian Republic (22 September 2019, 43°57′63.24″ N, 42°59′23.81″ E, 1905 m a.s.l.; Russia; flowering stage; 5 locations, 10 samples; voucher No PALE 005073-01–PALE 005073-10). The species were authenticated by Mikhail Goncharov, Ph.D. Biology (Saint Petersburg State Chemical and Pharmaceutical University, Saint Petersburg, Russia). Samples of the *V. arctostaphylos* L. leaves were collected from an alpine meadow in the Republic of Adygea, Khamyshki village, Maykop District (26 September 2020, 44°02′341″ N, 40°10′035″ E, 698 m a.s.l.; Russia; flowering stage; 5 locations, 10 samples; voucher No PALE 005082-01–PALE 005082). The species were authenticated by Dmitryi Shilnikov, Ph.D. Biology (Ecological and Botanical Station “Pyatigorsk”, Botanical Institute, Russian Academy of Sciences, Pyatigorsk, Russia). Plant material was dried at 40 °C (ventilated heat oven; 8–10 days) and stored at 3–4 °C before analysis. Selected chemicals were purchased from Sigma–Aldrich (St. Louis, MO, USA): acetonitrile for HPLC (Cat. No. 34851, ≥99.9%); formic acid (Cat. No. F0507, ≥95%); 4-*O*-caffeoylquinic acid (CAS 905-99-7, Sigma-Aldrich No. 65969, ≥98%); 5-*O*-caffeoylquinic acid (CAS 906-33-2, Sigma-Aldrich No. 94419, ≥98%); caffeic acid (CAS 331-39-5, Sigma-Aldrich No. C0625, ≥98%); 4,5-di-*O*-caffeoylquinic acid (CAS 14535-61-3, Sigma-Aldrich No. PHL80427, ≥95%); rutin (CAS 207671-50-9, Sigma-Aldrich No. R5143, ≥94%); hyperoside (CAS 482-36-0, Sigma-Aldrich No. 83388, ≥97%); isoquercitrin (CAS 482-35-9, Sigma-Aldrich No. 16,654, ≥98%); guaiaverin (CAS 22255-13-6, Sigma-Aldrich No. 94821, ≥95%); avicularin (CAS 572-30-5, Sigma-Aldrich No. PHL80361, ≥95%); quercitrin (CAS 849061-97-8, Sigma-Aldrich No. 337951, ≥95%); quercetin-3-*O*-(6″-*O*-acetyl)-glucoside (CAS 54542-51-7, No.1099, ≥85%, Extrasynthese, Lyon, France).

### 2.2. Plant Extract Preparation

Plant extract for HPLC analysis was prepared using 200 mg sample of milled material (particle size 0.125 μm) treated by 2 mL of 70% ethanol and sonication (ultrasonic bath, 30 min, 50 °C, ultrasound power 100 W, frequency 35 kHz; ×3). The ethanolic extract was centrifuged at 6000 × *g* (10 min), filtered through 0.22-μm syringe filters into a measuring flask (10 mL) and the final volume was filled up to 10 mL. The final extract was stored at 2 °C before analysis. Plant extract for the pharmacological study was prepared using the same procedure from 150 g-portion of material and drying in a vacuum oven. The total yields of *V. myrtillus* and *V. arctostaphylos* extracts were 49.3% and 40.1% of plant material weight, respectively.

### 2.3. High-Performance Liquid Chromatography with Photodiode Array Detection and Electrospray Ionization Triple Quadrupole Mass Spectrometric Detection (HPLC–PDA–ESI–QQQ–MS) 

High-performance liquid chromatography with photodiode array detection and electrospray ionization triple quadrupole mass spectrometric detection (HPLC–PDA–ESI–QQQ–MS) with LC-20 Prominence liquid chromatograph coupled with an SPD-M30A photodiode array detector, LCMS 8050 triple-quadrupole mass spectrometer and GLC Mastro C_18_ column (2.1 × 150 mm, 3 μm; all Shimadzu, Columbia, MD, USA) were used for the profiling and quantification of the plant extracts, as described previously [31]. The similarity of retention time, ultraviolet and mass spectral data was used for the identification of metabolites after comparison with the reference standards and literature data. The metrics of calibration curves as correlation coefficient (r^2^), standard deviation (S_YX_), limits of detection (LOD), limits of quantification (LOQ), and linear range were found for calibration solution (1, 10, 25, 50, 100 µg/mL) of 11 reference standards (4-*O*-caffeoylquinic acid, 5-*O*-caffeoylquinic acid, caffeic acid, 4,5-di-*O*-caffeoylquinic acid, rutin, hyperoside, isoquercitrin, guaiaverin, avicularin, quercitrin, quercetin-3-*O*-(6″-*O*-acetyl)-glucoside) using the known method [32] (Table 1).

### 2.4. Neuroprotective Activity

#### 2.4.1. Animals

The study was used 100 male Wistar rats, 180–200 g weight. The animals were purchased from the laboratory animal nursery “Rappolovo” (Russia, Leningrad region) and during the experiment were kept in polypropylene boxes by 5 rats in controlled conditions of detention: air temperature 20 ± 20 °C, humidity—55–65%, daily cycle 12 h a day/12 h a night. The treatment of laboratory animals and their maintenance met the requirements of Directive 2010/63/EU of the European Parliament and of the Council of the European Union of 22 September 2010 for the protection of animals used for scientific purposes. The local Ethics committee (Protocol of meeting No. 10 of 20 August 2021) approved the research concept.

#### 2.4.2. Brain Ischemia Model

Permanent focal cerebral ischemia was reproduced by right sided thermocoagulation of the middle cerebral artery (MCA). In this research, the concept irreversible technique was used. Rats were anesthetized by chloral hydrate (350 mg/kg, intraperitoneal). After, the area from the right side of the eye was depilated, skin was cut, and soft tissues were pushed apart, exposing the process of the zygomatic bone, which was deleted. Next, a trepanation hole was made with a drill and the MCA was burned with a thermocoagulator under the place of its intersection with the olfactory tract. If possible, the anatomic structure of soft tissues was restored. The suture was processed with a 10% povidone-iodine solution [33].

#### 2.4.3. Study Design

Experimental animals were divided into 5 equal groups of 20 individuals (the deviation of the body weight of rats in one experimental group did not exceed 10%). The first group was sham operated (SO), to whose animals all sequential surgical manipulations were applied with the exception of arterial coagulation; negative control (NC)—animals with focal cerebral ischemia, but deprived of pharmacological support (this group received purified water throughout the study); EGB761 is a group of rats that were treated with a reference—standardized extract of *Ginkgo biloba* (EGB761; obtained from Hunan Warrant Pharmaceuticals, Changsha, China) at a dose of 35 mg/kg [34]; VM is a group of animals that received extract from *V. myrtillus*; VA is a group of animals that received extract from *V. arctostaphylos*. The administered dose for the studied extracts was selected on the basis of previous studies and amounted to 50 mg/kg. The referent and the studied extracts were administered for 3 days after ischemia, orally, once a day. On the 4th day of the experiment, a change in the level of local cerebral blood flow was evaluated in rats, after which the animals were decapitated and the brain was extracted, in the tissue of which the change in the necrosis zone was evaluated (in 10 rats from the group). The activity of superoxide dismutase, cytochrome-c oxidase and succinate dehydrogenase was determined in the remaining 10 individuals from the group. Changes in the concentration of 2-thiobarbituric acid active substances (TBARS) in brain tissue were also evaluated. All tests were performed once for each animal. A total of 20 tests were performed for one experimental group.

#### 2.4.4. Cerebral Blood Flow Evaluation

The average systolic velocity of cerebral blood flow was recorded in the of the MCA area, for which a trepanation hole was made in this animal’s brain region. Rats were anesthetized by chloral hydrate. The average systolic velocity was recorded using an ultrasound Doppler device, a sensor USOP-010-01 with a work frequency of 25 MHz and a MM-D-K-Minimax Doppler v.1.7. (Saint Petersburg, Russia) software for Windows, using mathematical processing of the fast Fourier transform signal to transform the color spectrum of the blood flow distribution [35].

#### 2.4.5. Biomaterial Sampling and Preparation

Animal brains were used as biomaterial for analysis. The brain was homogenized in a mechanical homogenizer Potter type in a cold system of buffer solutions that consist of 1 mM EGTA, 215 mM mannitol, 75 mM sucrose, 0.1% bovine serum albumin solution with a 20 mM HEPES. The pH of buffer solution was 7.2. To obtain a primary homogenate, the ratio of tissue mass/volume of buffer solution 1:7 was used. Primary brain homogenate was separated into two parts. The first of them was used to determine the concentration of TBARS. The second part of the homogenate was used to determine superoxide dismutase, cytochrome c oxidase and succinate dehydrogenase activity. For that, homogenate was centrifuged 2 min at 1100× *g* acceleration. The supernatant was moved to Eppendorf-type test tubes and a percoll solution 10% concentration was layered, after which mixture was re-centrifuged at 18,000× *g* for 10 min. The secondary supernatant was deleted, the precipitate was resuspended in a buffer solution and re-centrifuged at 10,000× *g* for 5 min 

#### 2.4.6. Evaluation of Necrosis Zone

The size of the necrosis area of the brain was determined by the triphenyl tetrazolium method (n = 10 from each group). The brain was extracted from skull, the cerebellum was cut off. After, the hemispheres were separated. Both hemispheres were weighed. Next, the hemispheres were separately homogenized in PBS and placed in test-tubes with 10 mL of 1% triphenyltetrazolium chloride solution in a PBS with pH 7.4. The test-tubes were placed in a water bath for 20 min at a temperature of 37 ° C. Next, the probes were centrifuged in the mode of 3000 RCF/10 min and the resulting supernatant was removed. A total of 3 mL of phosphate buffer and 3 mL of cooled chloroform were added to the precipitate. Shaken for 2 min. Chloroform extract of formazan was obtained for 15 min at 4 ° C, shaking the mixture every 5 min for 30 s. The optical density (492 nm) against pure chloroform was centrifuged and measured. The necrosis zone area was expressed as a percentage of the total mass of the hemispheres and was calculated by formula:

$$x=100-\frac{\varepsilon_{1}M_{1}+\varepsilon_{2}M_{2}}{\varepsilon_{1}\left(M_{1}+M_{2}\right)}100\%,$$

where *x* is the size of the necrosis zone as a percentage of the total mass of the brain; *ε*_1_ is the optical density of the sample with an intact hemisphere; *ε*_2_ is the optical density of the sample with a damaged hemisphere; *M*_1_ is the mass of the intact hemisphere; *M*_2_ is the mass of the damaged hemisphere.

### 2.5. Antioxidant Potential

#### 2.5.1. Thiobarbituric Acid Reactive Substances (TBARS) Evaluation

The concentration of TBARS was evaluated in the brain homogenate by spectrophotometric method. The principle of the method is based on the spectrophotometric (at 532 nm) determination of colored products of the condensation reaction between unsaturated aldehydes and 2-thiobarbituric acid. In this case, the absorbance of the solution is correlated to the concentration of malondialdehyde. The amount of TBARS was calculated by the malondialdehyde molar extinction coefficient (1.56 × 10^5^ l × mol^−1^ × cm^−1^) value. The obtained results were expressed in nmol/mg protein. Protein content was determined by the Bradford method. Absorbance was recorded on Infinite F50 reader (Tecan Austria GmbH, Grödig, Austria) [36].

#### 2.5.2. Superoxide Dismutase (SOD) Activity 

The activity of SOD was estimated by the xanthine-xanthine oxidase method. Principal of a method is based on the determination of products of reaction of the dismutation of the superoxide radical that a was formed during the complex oxidation and reduction of xanthine and 2-(4-iodophenyl)-3-(4-nitrophenol)-5-phenyltetrazolium chloride, respectively. The reaction mixture consisted of 0.05 mM of xanthine; 0.025 mM of 2-(4-iodophenyl)-3-(4-nitrophenol)-5-phenyltetrazolium chloride; 0.94 mM of EDTA, 80 U/L of xanthine oxidase, 40 mM of CAPS (N-cyclohexyl-3-aminopropanesulfonic acid) buffer. The optical density of the mixture was estimated at 505 nm. SOD activity was expressed in units/protein mg. Protein content was determined by the Bradford method. Absorbance was recorded on Infinite F50 reader (Tecan Austria GmbH, Grödig, Austria) [37].

#### 2.5.3. Succinate Dehydrogenase (SDH) Activity 

The SDH activity was evaluated by spectrophotometric method which based on the reaction of the reduction of dichlorophenolindophenol that was catalyzed by succinate. To inhibit a mitochondrial complex I activity rotenone was added to the analyzed mixture. Absorbance of the probes was estimated at 600 nm [23]. During the analysis, standard kits from Abcam (ab110217/MS643) were used. Absorbance was recorded on Infinite F50 reader (Tecan Austria GmbH, Grödig, Austria) [38].

#### 2.5.4. Cytochrome-C-Oxidase (COX) Activity 

The COX activity was evaluated in the reaction of cytochrome C (II) oxidation when KCN was added in an incubation mixture. Absorbance was estimated at 500 nm (24). Standard kits from Abcam (ab110217/MS643) were used in the analysis. Absorbance was recorded on Infinite F50 reader (Tecan Austria GmbH, Grödig, Austria) [39].

### 2.6. Statistical Analysis

Statistical analysis of chemical data was performed by one-way analysis of variance, and the significance of the mean difference was determined by Duncan’s multiple range test. Differences at *p* < 0.05 were considered statistically significant. The results are presented as mean values ± standard deviations (S.D.). The linear regression analysis and generation of calibration graphs were conducted using Advanced Grapher 2.2 (Alentum Software Inc., Ramat-Gan, Israel). The statistical analysis of the results of neuroprotective activity evaluation was processed by using the STATISTICA 6.0 software (Dell Technologies Inc., Round Rock, TX, USA). The obtained data were expressed as M ± SEM (mean ± standard error of the mean). The normality was assessed by the Shapiro–Wilk test, the uniformity of variance was estimated by the Leven’s test. The ANOVA with post hoc Newman-Keusle test was used in post-processing analysis. A critical level of significance was *p* < 0.05.

## 3. Results

### 3.1. Phenolic Compounds of V. myrtillus and V. arctostaphylos Leaves

**Figure 2:**
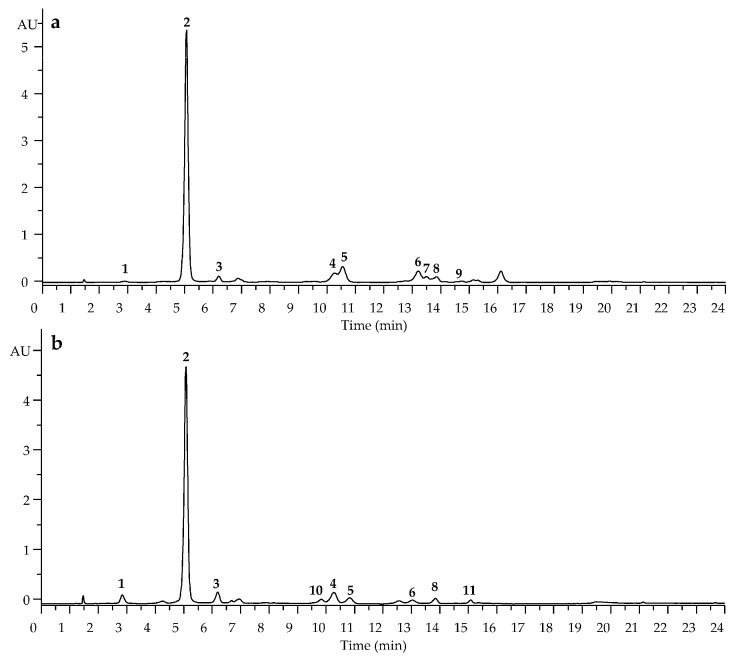

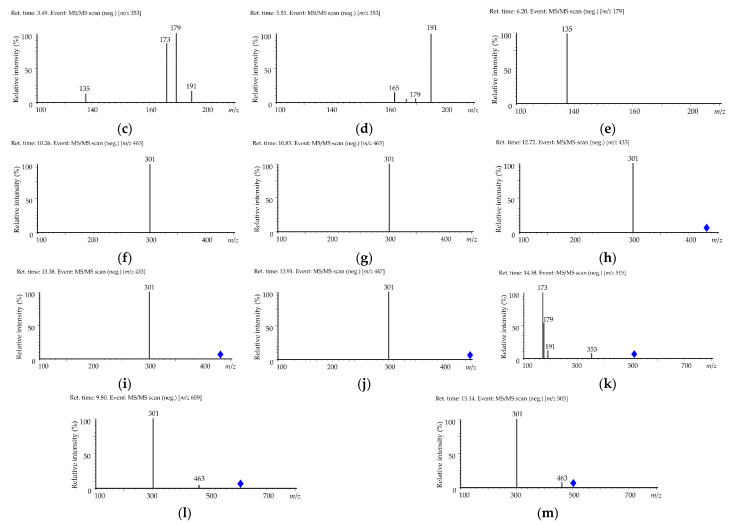
High-performance liquid chromatography with photodiode array detection (HPLC-PDA) chromatograms (330 nm) of leaf extracts of *V. myrtillus* (**a**) and *V. arctostaphylos* (**b**) and mass spectra of compounds **1** (**c**), **2** (**d**), **3** (**e**), **4** (**f**), **5** (**g**), **6** (**h**), **7** (**i**), **8** (**j**), **9** (**k**), **10** (**l**), and **11** (**m**). Compounds are numbered as listed in Table 2. Blue square indicates location of [M − H]^−^ ion.

Nine phenolic compounds, including the four phenolic acids of 4-*O*-caffeoylquinic, 5-*O*-caffeoylquinic, caffeic, and 4,5-di-*O*-caffeoylquinic acids as well as five flavonoid quercetin glycosides (quercetin-3-*O*-galactoside, quercetin-3-*O*-glucoside, quercetin-3-*O*-arabinopyranoside, quercetin-3-*O*-arabinofuranoside, quercetin-3-*O*-rhamnoside), were identified in *V. myrtillus* leaves (Figure 2, Table 2).

**Figure 3:**
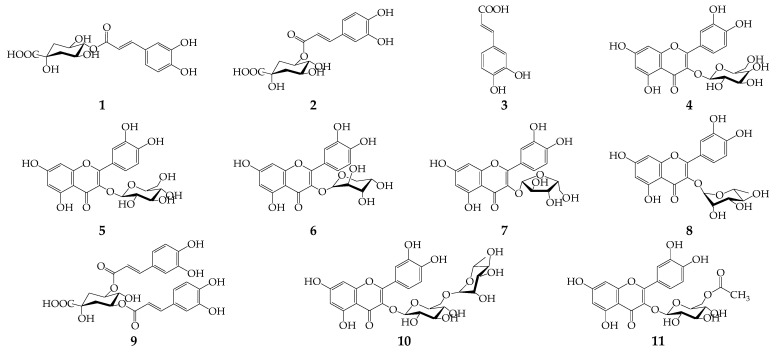
Compounds **1**–**11** found in leaf extracts of *V. myrtillus* and *V. arctostaphylos*.

Ten compounds of phenolic origin were found in *V. arctostaphylos* leave extracts, among them three phenolic acids (4-*O*-caffeoylquinic, 5-*O*-caffeoylquinic, and caffeic acids) and six quercetin glycosides [quercetin-3-*O*-rutinoside, quercetin-3-*O*-galactoside, quercetin-3-*O*-glucoside, quercetin-3-*O*-arabinopyranoside, quercetin-3-*O*-rhamnoside and quercetin-3-*O*-(6″-acetyl)-glucoside] (Figure 3).

Caffeoylquinic acids and caffeic acid, the known metabolites of *V. myrtillus* leaves [40], were also found in *V. arctostaphylos* leaves of Georgian origin [41]. In a previous study, the basic quercetin glycosides in *V. myrtillus* leaves from Finland were rutin, hyperoside, guaiaverin, avicularin, and quercitrin [42], and the only known flavonoid in *V. arctostaphylos* leaves is quercetin [43]. Quercetin 3-*O*-(6″-acetyl)-glucoside was found in *V. myrtillus* leaves for the first time as well as quercetin glycosides, which were newly discovered in *V. arctostaphylos* leaves. 

Results of the quantification of compounds **1**–**11** in *V. myrtillus* and *V. arctostaphylos* leaf extracts demonstrated high contents of 5-*O*-caffeoylquinic acid (226.85 mg/g in *V. myrtillus*, 105.32 mg/g in *V. arctostaphylos*), quercetin 3-*O*-glucoside (12.02 mg/g in *V. myrtillus*), and quercetin 3-*O*-galactoside (12.02 mg/g in *V. arctostaphylos*) (Table 3). Earlier, chlorogenic acid was detected as the main phenolic compound in the *V. myrtillus* leaves (approximately 52–84% of the total amount of phenols) [44,45], and quercetin and kaempferol derivatives were the predominant phenolic groups (39.2%) [46]. The water–alcoholic extract of *V. arctostaphylos* leaves from Iran contained chlorogenic acid and flavonoids at 97.2 mg/g and 2.2–22.4 mg/g, respectively [15,47].

### 3.2. Neuroprotective Activity of V. myrtillus and V. arctostaphylos Leaf Extracts

**Figure 4:**
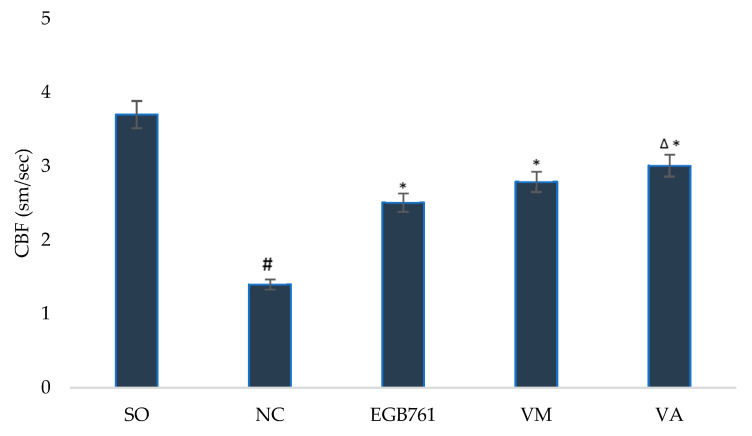
The effect of *V. myrtillus* and *V. arctostaphylos* leaf extracts on the cerebral hemodynamics in rats with cerebral ischemia. Experimental groups: SO—sham-operated animals; NC—negative control animals; EGB761—EGB761 reference remedy group; VM—*V. myrtillus* group; VA—*V. arctostaphylos* group. Hashtag (#) indicates significant difference (*p* < 0.05) vs. the SO group; asterisk (*) indicates significant difference (*p* < 0.05) vs. the NC group; delta (Δ) indicates significant difference (*p* < 0.05) vs. the EGB761 group.

The study of neuroprotective activity of extracts from *V. myrtillus* and *V. arctostaphylos* showed that their administration to animals in the post-ischemic period contributed to an increase in the level of cerebral blood flow (Figure 4) compared to that of untreated rats by 99.3% (*p* < 0.05) and 115.0% (*p* < 0.05), respectively, while the use of the EGB761 reference increased this indicator by 79.3% (*p* < 0.05). Of note, in animals that received extraction from *V. arctostaphylos*, the level of cerebral blood flow was higher in the rats injected with the reference by 19.9% (*p* < 0.05).

**Figure 5:**
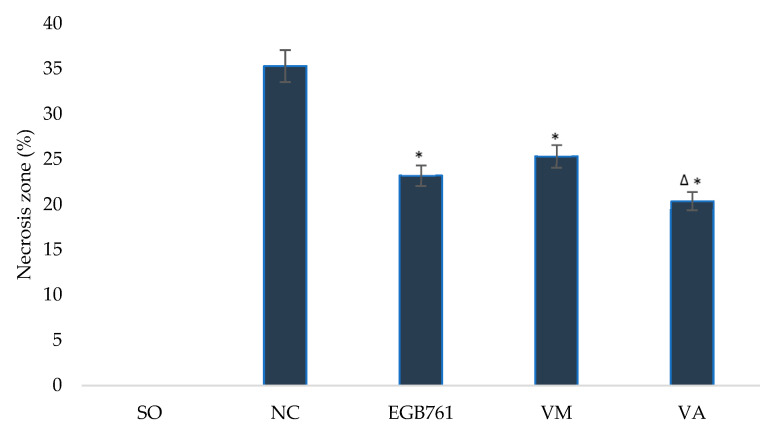
The effect of *V. myrtillus* and *V. arctostaphylos* leaf extracts on the necrosis zone in rats with cerebral ischemia. Experimental groups: SO—sham-operated animals; NC—negative control animals; EGB761—EGB761 reference remedy group; VM—*V. myrtillus* group; VA—*V. arctostaphylos* group. Asterisk (*) indicates significant difference (*p* < 0.05) vs. the NC group; delta (Δ) indicates significant difference (*p* < 0.05) vs. the EGB761 group.

Additionally, the use of the studied extracts led to a decrease in the brain necrosis zone (Figure 5), while against the background of the administration of the extract from *V. myrtillus,* this indicator decreased by 28.2% (*p* < 0.05), and for *V. arctostaphylos*, it decreased by 42.2% (*p* < 0.05). At the same time, the use of the reference contributed to a decrease in the brain necrosis zone by 34.3% (*p* < 0.05), which was higher than the same indicator for the group of animals that were injected with extraction from *V. arctostaphylos* by 12.1% (*p* < 0.05).

### 3.3. Antioixdant Potential of V. myrtillus and V. arctostaphylos Leaf Extracts

The study of changes in the antioxidant balance of brain tissue with the administration of the studied extracts showed that the use of extracts from *V. myrtillus* and *V. arctostaphylos* contributed to an increase in the activity of SOD by 4.1% (*p* < 0.05) and 76.4% (*p* < 0.05), respectively, and reduced the content of TBARS by 55.1% (*p* < 0.05) and 64.3% (*p* < 0.05), respectively (Table 4). 

At the same time, the use of EGB761 contributed to an increase in SOD activity by 51.7% (*p* < 0.05) and a decrease in TBARS by 46.9% (*p* < 0.05), which was statistically significantly lower than similar indicators of animals receiving extraction from *V. arctostaphylos*. Analysis of changes in the activity of mitochondrial enzymes showed that when extracts from *V. myrtillus* L. and *V. arctostaphylos* were used, as well as the reference, the activity of COX increased (relative to the NC group of rats) by 23.8% (*p* < 0.05), 42.9% (*p* < 0.05), and 19.0% (*p* < 0.05), respectively, whereas SDH activity increased by 36.4% (*p* < 0.05), 45.5% (*p* < 0.05), and 90.9% (*p* < 0.05). Of note, in rats injected with the extract from *V. arctostaphylos*, the activities of both COX and SDH were higher than in animals treated with the reference and extract from *V. myrtillus* (*p* < 0.05).

## 4. Discussion

A detailed study of plant metabolites opens possibilities for selection of precise and accurate methods to include in pharmacopeial practice. The results obtained in this study show that the major and marker compound for both *Vaccinium* species was 5-*O*-caffeoylquinic acid (chlorogenic acid). In predicting the pharmacological activity, phenolic acids are mostly represented by chlorogenic acid isomers (4-*O*-caffeoylquinic and 5-*O*-caffeoylquinic acids). Moreover, the main aglycone of flavonoids is quercetin, and its glycosides are presented by monosides (quercetin-3-*O*-galactoside, quercetin-3-*O*-glucoside, quercetin-3-*O*-arabinopyranoside, quercetin-3-*O*-arabinofuranoside, quercetin-3-*O*-rhamnoside, and quercetin-3-*O*-(6″-acetyl)-glucoside) and by only one bioside (quercetin-3-*O*-rutinoside). Chlorogenic acids and quercetin glycosides are known neuroprotective agents. Thus, chlorogenic acid shows a protective effect on the nitrosative stress induced by sodium nitroprusside [48]. In vivo, chlorogenic acid decreased the infarct area in the hypoxic–ischemic brains of rats [49]. Additionally, chlorogenic acid may be a potential agent to treat Parkinson’s disease in the apoptosis-mediated neuronal senescence of dopaminergic neurons [50]. The neuroprotective effects of quercetin may be used in the treatment of Alzheimer’s disease via its antioxidant properties and providing an inhibitory effect of fibril formation of amyloid-β aggregates [51]. These effects are useful in the therapy of pediatric neurological diseases such as central nervous system tumors, autism, and hyperactivity disorder [52]. Quercetin and rutin show neuroprotective effects in the cerebral ischemia model in rats by its 4-oxo group and 2,3-double bond in the C ring [53]. Thus, the potential neuroprotective effects of *Vaccinium* extracts may be related to the significant amount of these phenolics in the *Vaccinium* leaves.

Increasingly, natural remedies are being used as adjuvant neuroprotective therapy for cerebrovascular disorders [54]. The neuroprotective effects of traditional Chinese medicine (for example, ginseng preparations, as well as *Scutellaria baicalensis*, *Withania somnifera*, *Phyllanthus emblica*, and *Viscum album* [55]) are widely known. It has been established that the use of phytomedicines has a pleiotropic effect on brain tissue, while eliminating almost all main pathogenetic pathways of the “ischemic cascade”: neuroinflammation, apoptosis, energy deficiency, glutamate-calcium excitotoxicity, hyperlactatemia, and acidosis [55]. However, as a rule, remedies based on phytocompositions are known as effective antioxidants [55,56].

The concept of phytotherapeutic intervention in ischemic stroke is currently being actively studied; however, there is no doubt that this approach is only auxiliary and cannot fully replace the “gold standard” of treatment of ischemic brain damage—effective thrombolysis [57]. The rapidly developing direction of phytotherapeutic neuroprotection makes it necessary to search for new drugs that meet the basic postulates of this area of medicine and pharmacology [58]. In this work, possible neuroprotective properties of extracts obtained from two species of representatives of the heather family, *V. myrtillus* and *V. arctostaphylos*, were studied. Blueberry-based products are primarily known as effective biologically active additives with pronounced antioxidant activity and tropicity in relation to blood vessels and the cardiovascular system as a whole, which has made it possible to use these products in the correction of vascular complications of diabetes mellitus, atherosclerosis, and glaucoma [58]. At the same time, a similar therapeutic profile of extracts from blueberries suggests the presence of neuroprotective activity. During the study, it was found that the administration of extracts obtained from *V. myrtillus* and *V. arctostaphylos* to animals with focal ischemia contributed to the improvement of cerebral hemodynamics in the post-ischemic period, which may be associated with the restoration of the function of collateral blood supply to the ischemic zone. A decrease in the necrotic focus was also shown in rats receiving the analyzed extracts compared with untreated animals. Because one of the significant mechanisms of ischemic brain damage is the development of oxidative stress, we studied the effect of extracts from blueberries on changes in the parameters characterizing the development of this pathological process—the activity of superoxide dismutase and the final concentration of peroxidation products represented by TBARS [36]. As a result, the introduction of extracts from *V. myrtillus* and *V. arctostaphylos* increased the activity of superoxide dismutase and reduced the intensity of free radical lipid oxidation reactions. An important aspect of brain ischemia is a violation of the energy state of cells with a sharp drop in ATP synthesis and the induction of irreversible processes in the form of necroptosis or apoptosis [59]. In this regard, the effect of extracts from *V. myrtillus* and *V. arctostaphylos* on the activity of two integral enzymatic indicators of the metabolic status of the cell, cytochrome c oxidase and succinate dehydrogenase, was evaluated. The administration of the analyzed extracts led to an increase in the activity of these enzymes, and the effect of the administration of the extract obtained from *V. arctostaphylos* exceeded that of the reference, the classical neuroprotective herbal remedy *Ginkgo biloba* extract (EGB761). Thus, based on the overall results of the set of pharmacological tests of extracts from *V. myrtillus* and *V. arctostaphylos*, it can be assumed that these objects have neuroprotective activity, which can be realized through antioxidant and metabolic action. At the same time, a more promising therapeutic agent is the extraction from *V. arctostaphylos*, the use of which led to a statistically significantly better pharmacological response than the introduction of the reference and extract obtained from *V. myrtillus.*

## Figures and Tables

**Table 1:** Regression equations, correlation coefficients (*r*^2^), standard deviation (*S*_YX_), limits of detection (LOD), limits of quantification (LOQ), and linear ranges for 11 reference standards.

Compound	Regression Equation ^a^		*r* ^2^	*S*_YX_	LOD/LOQ (µg/mL)	Linear Range (µg/mL)
	a	b × 10^6^				
4-*O*-Caffeoylquinic acid	0.162	−0.011	0.9973	1.83 × 10^−2^	0.37/1.12	2–100
5-*O*-Caffeoylquinic acid	0.150	−0.010	0.9982	1.67 × 10^−2^	0.36/1.11	2–100
Caffeic acid	0.189	−0.017	0.9988	1.26 × 10^−2^	0.22/0.67	1–100
Quercetin 3-*O*-rutinoside (rutin)	0.085	−0.062	0.9873	3.89 × 10^−2^	1.51/4.57	5–100
Quercetin 3-*O*-galactoside (hyperoside)	0.090	−0.052	0.9891	3.52 × 10^−2^	1.29/3.91	4–100
Quercetin 3-*O*-glucoside (isoquercitrin)	0.098	−0.053	0.9889	3.37 × 10^−2^	1.14/3.43	4–100
Quercetin 3-*O*-arabinopyranoside (guaiaverin)	0.121	−0.031	0.9953	2.59 × 10^−2^	0.70/2.14	3–100
Quercetin 3-*O*-arabinofuranoside (avicularin)	0.114	−0.026	0.9927	2.80 × 10^−2^	0.81/2.46	3–100
Quercetin 3-*O*-rhamnoside (quercitrin)	0.109	−0.043	0.9912	3.16 × 10^−2^	0.96/2.90	3–100
Quercetin 3-*O*-(6″-acetyl)-glucoside	0.095	−0.050	0.9880	3.28 × 10^−2^	1.14/3.45	4–100
4,5-Di-*O*-caffeoylquinic acid	0.163	−0.016	0.9975	1.93 × 10^−2^	0.39/1.18	2–100

^a^ Regression equation: y = a∙x + b.

**Table 2:** Retention times (t), ultraviolet (UV), and mass spectral (ESI-MS, MS/MS) data of compounds **1**–**11** were found for leaf extracts of *V. myrtillus* and *V. arctostaphylos*.

No	t, min	Compound *	UV, λ_max_, nm	ESI-MS, [M − H]^−^, *m*/*z*	MS/MS, *m*/*z*
**1**	3.49	4-*O*-Caffeoylquinic acid	322	353	[353]: 191, 179, 173, 135
**2**	5.51	5-*O*-Caffeoylquinic acid	322	353	[353]: 191, 179, 165
**3**	6.20	Caffeic acid	320	179	[179]: 135
**4**	10.26	Quercetin 3-*O*-galactoside (hyperoside)	255, 267, 355	463	[463]: 301
**5**	10.83	Quercetin 3-*O*-glucoside (isoquercitrin)	255, 267, 356	463	[463]: 301
**6**	12.72	Quercetin 3-*O*-arabinopyranoside (guaiaverin)	255, 267, 354	433	[433]: 301
**7**	13.38	Quercetin 3-*O*-arabinofuranoside (avicularin)	255, 267, 354	433	[433]: 301
**8**	13.91	Quercetin 3-*O*-rhamnoside (quercitrin)	255, 267, 352	447	[447]: 301
**9**	14.58	3,5-Di-*O*-caffeoylquinic acid	322	515	[515]: 353, 191, 179, 173
**10**	9.80	Quercetin 3-*O*-rutinoside (rutin)	255, 267, 355	609	[609]: 463, 301
**11**	15.14	Quercetin 3-*O*-(6″-acetyl)-glucoside	256, 268, 351	505	[505]: 463, 301

* Identification was performed by comparison of obtained retention times, UV, MS, and MS/MS with reference standards.

**Table 3:** Contents of compounds **1**–**11** in leaf extracts of *V. myrtillus* and *V. arctostaphylos* in mg/g of dry weight ± S.D.

Compound	*V. myrtillus*	*V. arctostaphylos*
4-*O*-Caffeoylquinic acid	<0.01	8.01 ± 0.14
5-*O*-Caffeoylquinic acid	226.85 ± 5.21	105.32 ± 2.41
Caffeic acid	<0.01	4.29 ± 0.08
Quercetin 3-*O*-rutinoside (rutin)	<0.01	1.46 ± 0.03
Quercetin 3-*O*-galactoside (hyperoside)	4.69 ± 0.09	3.99 ± 0.06
Quercetin 3-*O*-glucoside (isoquercitrin)	12.02 ± 0.24	2.38 ± 0.05
Quercetin 3-*O*-arabinopyranoside (guaiaverin)	1.34 ± 0.02	1.29 ± 0.02
Quercetin 3-*O*-arabinofuranoside (avicularin)	<0.01	<0.01
Quercetin 3-*O*-rhamnoside (quercitrin)	2.77 ± 0.05	1.23 ± 0.02
Quercetin 3-*O*-(6″-acetyl)-glucoside	<0.01	0.70 ± 0.01
4,5-Di-*O*-caffeoylquinic acid	<0.01	<0.01

**Table 4:** Effect of *V. myrtillus* and *V. arctostaphylos* leaf extracts on activity of mitochondrial enzymes and TBARS in rats with cerebral ischemia.

Experimental Group	SOD, U/Protein mg	COX, U/Protein mg	SDH, U/Protein mg	TBARS, µM/Protein mg
Sham-operated animals	300.5 ± 7.1(29)	4.3 ± 0.0(47)	2.7 ± 0.062	2.4 ± 0.124
Negative control group	125.6 ± 9.5(41) ^#^	2.1 ± 0.0(14) ^#^	1.1 ± 0.097 ^#^	9.8 ± 0.663 ^#^
EGB761 reference group	190.5 ± 11.2(84) *	2.5 ± 0.1(17) *	1.6 ± 0.018 *	5.2 ± 0.541 *
*V. myrtillus* group	181.0 ± 10.9(37) *	2.6 ± 0.0(37) *	1.5 ± 0.09 *	4.4 ± 0.364 *
*V. arctostaphylos* group	221.5 ± 10.2(32) *	3.0 ± 0.0(58) * Δα	2.1 ± 0.071 * Δα	3.5 ± 0.193 *

Hashtag (#) indicates significant difference (*p* < 0.05) vs. sham-operated animals’ group; asterisk (*) indicates significant difference (*p* < 0.05) vs. negative control group; delta (Δ) indicates significant difference (*p* < 0.05) vs. the EGB761 reference group; alpha (α) indicates significant difference (*p* < 0.05) vs. the *V. myrtillus* group.

## Data Availability

Data are contained within the article.
